# Prevalence of the prion protein gene E211K variant in U.S. cattle

**DOI:** 10.1186/1746-6148-4-25

**Published:** 2008-07-14

**Authors:** Michael P Heaton, John W Keele, Gregory P Harhay, Jürgen A Richt, Mohammad Koohmaraie, Tommy L Wheeler, Steven D Shackelford, Eduardo Casas, D Andy King, Tad S Sonstegard, Curtis P Van Tassell, Holly L Neibergs, Chad C Chase, Theodore S Kalbfleisch, Timothy PL Smith, Michael L Clawson, William W Laegreid

**Affiliations:** 1 USDA, ARS, U. S. Meat Animal Research Center (USMARC), State Spur 18D, P.O. Box 166, Clay Center, NE 68933, USA; 2 USDA, ARS, National Animal Disease Center (NADC), 2300 Dayton Avenue, Ames, IA 50010, USA; 3 USDA, ARS, Beltsville Agricultural Research Center (BARC), 10300 Baltimore Avenue, Beltsville, MD 20705, USA; 4 Washington State University, Department of Animal Sciences, P.O. Box 646353, Pullman, WA 99164, USA; 5 USDA, ARS, SubTropical Agricultural Research Station (STARS), 22271 Chinsegut Hill Road, Brooksville, FL 34601-4672, USA; 6 University of Louisville, Center for Genetics and Molecular Medicine, 580 South Preston Street, Louisville, KY 40202, USA; 7 IEH Laboratories & Consulting Group, 15300 Bothell Way NE, Lake Forest Park, WA 98155 USA; 8 University of Illinois, Department of Veterinary Pathobiology, 2001 S. Lincoln Avenue, Urbana, IL 61802, USA

## Abstract

**Background:**

In 2006, an atypical U.S. case of bovine spongiform encephalopathy (BSE) was discovered in Alabama and later reported to be polymorphic for glutamate (E) and lysine (K) codons at position 211 in the bovine prion protein gene *(Prnp*) coding sequence. A bovine E211K mutation is important because it is analogous to the most common pathogenic mutation in humans (E200K) which causes hereditary Creutzfeldt – Jakob disease, an autosomal dominant form of prion disease. The present report describes a high-throughput matrix-associated laser desorption/ionization-time-of-flight mass spectrometry assay for scoring the *Prnp *E211K variant and its use to determine an upper limit for the K211 allele frequency in U.S. cattle.

**Results:**

The K211 allele was not detected in 6062 cattle, including those from five commercial beef processing plants (3892 carcasses) and 2170 registered cattle from 42 breeds. Multiple nearby polymorphisms in *Prnp *coding sequence of 1456 diverse purebred cattle (42 breeds) did not interfere with scoring E211 or K211 alleles. Based on these results, the upper bounds for prevalence of the E211K variant was estimated to be extremely low, less than 1 in 2000 cattle (Bayesian analysis based on 95% quantile of the posterior distribution with a uniform prior).

**Conclusion:**

No groups or breeds of U.S. cattle are presently known to harbor the *Prnp *K211 allele. Because a carrier was not detected, the number of additional atypical BSE cases with K211 will also be vanishingly low.

## Background

Transmissible spongiform encephalopathies (TSE), or prion diseases, are fatal neurological disorders of humans and other mammals that are characterized by accumulation of an abnormal, protease-resistant isoform of the prion protein (PrP^TSE^) in the brain. In cattle, the largest disease outbreak was first recognized in 1986 in Great Britain and peaked in the early 1990's when the number of confirmed bovine spongiform encephalopathy (BSE) cases rose to more than 30,000 per year [1,2]. During this time, BSE transmission among cattle was caused primarily by feeding meat and bone meal derived from other BSE-affected cattle [3,4]. This so-called classical, or orally acquired BSE has since been identified in 24 additional countries around the world [5]. Consumption of beef from BSE-affected animals was implicated as the most likely cause of one human prion disease, variant Creutzfeldt-Jakob Disease (vCJD) [6-9]. However, after regulations were implemented to prevent BSE-contaminated tissues from entering the animal feed supplies and active BSE surveillance was increased, the number of BSE cases dropped dramatically [5]. This was followed by a corresponding reduction in vCJD cases [10].

An important outcome of intensive worldwide BSE surveillance has been the detection of atypical BSE in cattle. Atypical BSE cases may be distinguished from classical BSE by differences in: 1) distribution in the central nervous system, 2) molecular typing profile of PrP^TSE ^by Western blot, 3) distribution of cases over time, and 4) outcomes of transmission studies in animal models [11-14]. A striking feature of atypical BSE cases is their advanced age at time of detection. For example, the average age of atypical BSE cases is 12 years at the time of detection, compared to an average of 6 years for orally acquired BSE cases [1,15,16]. Although few atypical BSE cases have been identified worldwide (approximately 30), they are significant because of their possible link to sporadic CJD in humans (i.e., CJD with unknown origins) [15].

Recent evidence suggests that specific bovine prion protein gene (*Prnp*) variants may represent genetic risk factors for atypical BSE in older cattle. The first of two indigenous U.S. BSE cases (a 12-year-old, cream-colored, Brahman cross from a Texas farm in November 2004) was found to be homozygous for a particular *Prnp *haplotype associated with atypical BSE [17]. The second BSE case (an approximately 10-year-old, deep red-colored, crossbred beef cow from an Alabama farm in March 2006) had a previously unidentified, non-synonymous E211K mutation in the *Prnp *coding sequence (unpublished results, J.A. Richt and S.M. Hall). Genetic risk factors for TSE diseases are well known in human populations where more than 20 pathogenic *Prnp *mutations have been discovered in families with inherited prion diseases [18]. These are transmitted as autosomal dominant disorders and include familial CJD, Gerstmann-Sträussler-Scheinker disease, and fatal familial insomnia. Because both indigenous U.S. BSE cases have arisen without any known exposure to other infectious prion agents, the possibility remains that they represent a type of inherited BSE that occurs in older cattle with certain *Prnp *haplotypes.

**Figure 1:**
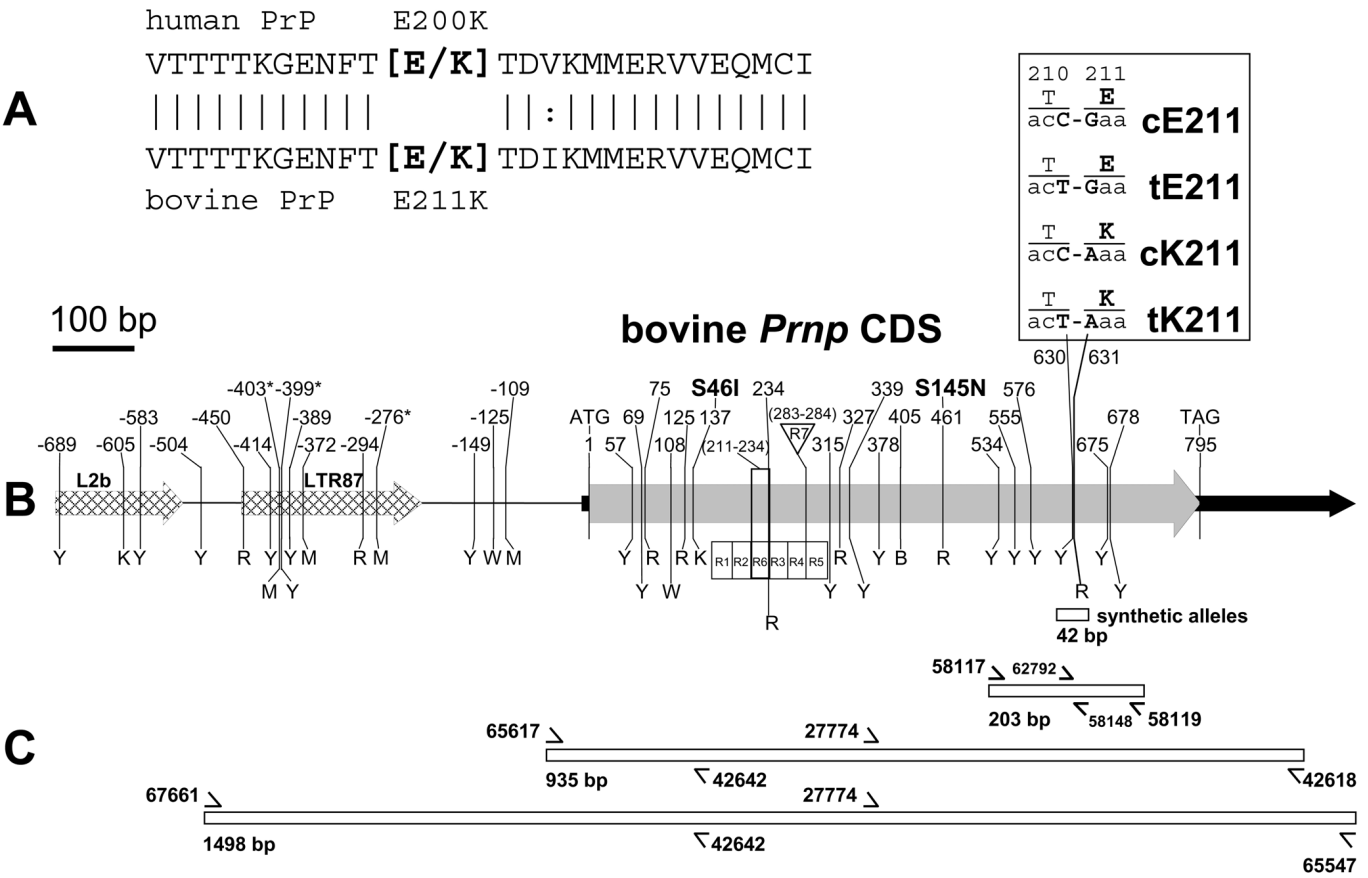
**Alignment of human and bovine PrP and physical maps of bovine *Prnp***. Panel A: Alignment of PrP sequences at human codon E200K and bovine codon E211K. Bovine codon 211 refers to the most common *Prnp *haplotype containing six octapeptide repeats. For those bovine haplotypes with five or seven octapeptide repeats, the equivalent codons would be numbered 203 and 219, respectively. Panel B: Physical map of bovine *Prnp *(1691 bp) showing the positions of known polymorphisms and the four possible haplotype sequences of codons 210 and 211 (abbreviations: cE211, acC-Gaa; tE211, acT-Gaa; cK211, acC-Aaa; tK211, acT-Aaa). The map features include: thick shaded arrow, coding sequence; black arrow, 5' and 3' untranslated regions of exon 3; and hatched arrow, bovine repetitive elements. The numbers above the vertical tick marks indicate the polymorphism position relative to the first base of the *Prnp *start codon (GenBank Accession number AY335912). The letters below the vertical tick marks are International Union of Biochemistry (IUB) ambiguity codes for SNPs in the sense direction (B = c/g/t, K = g/t, M = a/c, R = a/g, W = a/t, and Y = c/t). R1 through R7 refer to octapeptide repeats. R1 through R5 are octapeptide repeats orthologous to those in sheep. The previously reported non-synonymous polymorphisms at codons 46 (S46I) and 145 (S145N) are indicated at nt positions 137 and 461, respectively. The asterisks at nt positions -403, -399, and -276 denote polymorphisms in intron 2 that were not previously reported. Panel C: Amplicons for DNA sequencing or MALDI-TOF MS genotyping. Arrows denote positions of oligonucleotide primers used for amplification, sequencing, or primer extension and MALDI-TOF MS genotyping. The relative position of the 42-bp double stranded synthetic alleles is shown below positions 630 and 631 (i.e., below bovine codons 210 and 211).

The discovery of a *Prnp *E211K variant in an atypical BSE case is particularly remarkable because it is analogous to the most common pathogenic mutation in humans (E200K) which causes hereditary CJD (Figure 1A). In the human E200K mutation, the normal glutamate (E) codon GAG is replaced by a lysine (K) codon AAG. First reported in 1989, this human G to A transition at codon 200 has arisen independently at least four times in human history [19,20]. The Alabama atypical BSE case had a normal GAA (E) and a novel AAA (K) codon at position 211 in the prion gene (unpublished results, J.A. Richt and S.M. Hall). Investigators have not been able to identify the source of this apparent bovine G to A transition or the affected cow's herd of origin [21].

Knowledge of the distribution and frequency of *Prnp *E211K variants in cattle populations is critical for understanding and managing atypical BSE. The K211 allele had not previously been observed in a diverse sample of 311 full blood U.S. beef and dairy cattle [22-24]. However, it was not known whether the K211 allele was prevalent at a low frequency in commercially-produced crossbred cattle or was detectable in a more extensive sample of purebred cattle. To evaluate these possibilities, cattle from U.S. beef processing plants and registered purebred animals were genotyped at the *Prnp *locus. The present report describes a matrix-associated laser desorption/ionization-time-of-flight mass spectrometry (MALDI-TOF MS) genotype assay for accurate high-throughput scoring of the *Prnp *E211K variant and its use to determine an upper limit for the K211 allele prevalence in U.S. cattle.

## Results

### Scoring haplotypes and diplotypes of *Prnp *codons 210 and 211

The position of the E211K mutation is adjacent to that of a synonymous C/T polymorphism in codon 210. Thus it may be possible to observe four haplotype combinations of *Prnp *codons 210 and 211 (Figure 1B, haplotype abbreviations: cE211, tE211, cK211, and tK211). Moreover, ten diplotypes are possible when all paired combinations of four haplotypes are considered. To account for these ten possible paired haplotype combinations, homogeneous mass extension (hME) reactions were designed to generate both the sense and antisense allele-specific extension products with unique molecular masses (Table 1). Because most of the ten diplotypes had not been identified in cattle and thus could not be used as DNA controls, double-stranded synthetic DNA controls (42 bp) were used in their place (Table 1). MALDI-TOF MS analysis of the resulting extension products showed that the alleles were sufficiently resolved for accurate diplotype assignment (data not shown). Importantly, all possible K211 alleles were well resolved in both the sense and the antisense reactions, providing internal confirmation of the presence of any potential K211 allele. The predicted relative positions of all four haplotype alleles in their ten paired combinations are shown graphically in Additional file 1. These assays and their synthetic DNA controls provide the basis for accurate, high-throughput screening in cattle.

**Table 1:** Oligonucleotides for sequencing and genotyping *Prnp *alleles

Primer number^a^	DNA sequence^b^	Gene region	Orientation	Function	T_m _(°C)	STS length (bp)	Termination mixture	Mass (Daltons)	Codons 210/211^c^
67661	gac att aga atc act tcc ata gg	Intron 2, CDS, 3' UTR	Sense	Amplification, sequencing	58	1498	**-**^d^	**-**	**-**
65547	ata ctg agc taa cgg gac tt	Intron 2, CDS, 3' UTR	Antisense	Amplification, sequencing	58	1498	**-**	**-**	**-**
65617	gat ttt tac atg ggc ata tga	Intron 2, CDS, 3' UTR	Sense	Amplification, sequencing	60	985	**-**	**-**	**-**
42618	gcc aag ggt att agc ata ca	Intron 2, CDS, 3' UTR	Antisense	Amplification, sequencing	60	985	**-**	**-**	**-**
42642	cct gga ggc aac cgt tat	CDS	Sense	Sequencing	62	**-**	**-**	**-**	**-**
27774	ctc ctg cca cat gct tca t	CDS	Antisense	Sequencing	62	**-**	**-**	**-**	**-**
58117	tac agg cca gtg gat cag ta	CDS	Sense	Amplification	60	203	**-**	**-**	**-**
58119	gcc cct cgt tgg taa taa	CDS	Antisense	Amplification	60	203	**-**	**-**	**-**
58118	acg ttg gat gta cag gcc agt gga tca gta	CDS	Sense	Amplification for hME (with mass tag)	60	226	**-**	9311.10	**-**
58120	tgg acg ttg gat ggc ccc tcg ttg gta ata a	CDS	Antisense	Amplification for hME (with mass tag)	60	226	**-**	9582.20	**-**
58141	caccaagggggagaacttcac-C-G-aaactgacatcaagatgat	CDS	Sense	control template for cE211 allele	93	**-**	**-**	12958.40	**-**
58142	atcatcttgatgtcagttt-C-G-gtgaagttctcccccttggtg	CDS	Antisense	control template for cE211 allele	93	**-**	**-**	12868.30	**-**
64161	caccaagggggagaacttcac-T-G-aaactgacatcaagatgat	CDS	Sense	control template for tE211 allele	93	**-**	**-**	12973.50	**-**
64162	atcatcttgatgtcagttt-C-A-gtgaagttctcccccttggtg	CDS	Antisense	control template for tE211 allele	93	**-**	**-**	12852.30	**-**
58143	caccaagggggagaacttcac-C-A-aaactgacatcaagatgat	CDS	Sense	control template for cK211 allele	93	**-**	**-**	12942.40	**-**
58144	atcatcttgatgtcagttt-T-G-gtgaagttctcccccttggtg	CDS	Antisense	control template for cK211 allele	93	**-**	**-**	12883.30	**-**
58145	caccaagggggagaacttcac-T-A-aaactgacatcaagatgat	CDS	Sense	control template for tK211 allele	93	**-**	**-**	12957.50	**-**
58146	atcatcttgatgtcagttt-T-A-gtgaagttctcccccttggtg	CDS	Antisense	control template for tK211 allele	93	**-**	**-**	12867.30	**-**
62792	caa ggg gga gaa ctt cac	CDS	Sense	hME primer	58	**-**	ddA^e^	5557.60	**-**
**-**	caa ggg gga gaa ctt cac ca	CDS	Sense	CA allele analyte	-	**-**	ddA	6144.00^f^	**cK211**
**-**	caa ggg gga gaa ctt cac ta	CDS	Sense	TA allele analyte	-	**-**	ddA	6159.00^f^	**tK211**
**-**	caa ggg gga gaa ctt cac cga	CDS	Sense	CGA allele analyte	-	**-**	ddA	6473.20^f^	**cE211**
**-**	caa ggg gga gaa ctt cac tga	CDS	Sense	TGA allele analyte	-	**-**	ddA	6488.20^f^	**tE211**
58148	atc atc ttg atg tca gtt t	CDS	Antisense	hME primer	49	**-**	ddC/ddG^e^	5783.80	**-**
**-**	atc atc ttg atg tca gtt tc	CDS	Antisense	C allele analyte	-	**-**	ddC/ddG	6057.00	**nE211**
**-**	atc atc ttg atg tca gtt ttg	CDS	Antisense	TG allele analyte	-	**-**	ddC/ddG	6401.20	**cK211**
**-**	atc atc ttg atg tca gtt tta g	CDS	Antisense	TAG allele analyte	-	**-**	ddC/ddG	6714.40	**tK211**

^a^USMARC primer number; GenBank Accession number AY335912

^b^Primer sequences are listed 5' to 3'; primers 58118 and 58120 are analogous to primers 58117 and 58119, respectively, but with additional nucleotides on the 5' end (5'-acgttggatg-3' and 5'-tggacgttggatg-3', respectively) to shift their masses out of the analyte range.

^c^nE211 refers to either cE211 or tE211 since the genotype for this analyte is ambiguous in the antisense direction.

^d^Not applicable.

^e^Mixture also contains the complement dNTPs.

^f^Although a conservative hME assay design includes 40 Dalton spacing between analytes, only 15 Dalton spacing was possible in this case. Nevertheless, the typical instrument resolution of approximately 4 Daltons is sufficient to resolve these analytes.

### The prevalence of *Prnp *K211 allele in U.S. cattle

**Figure 2:**
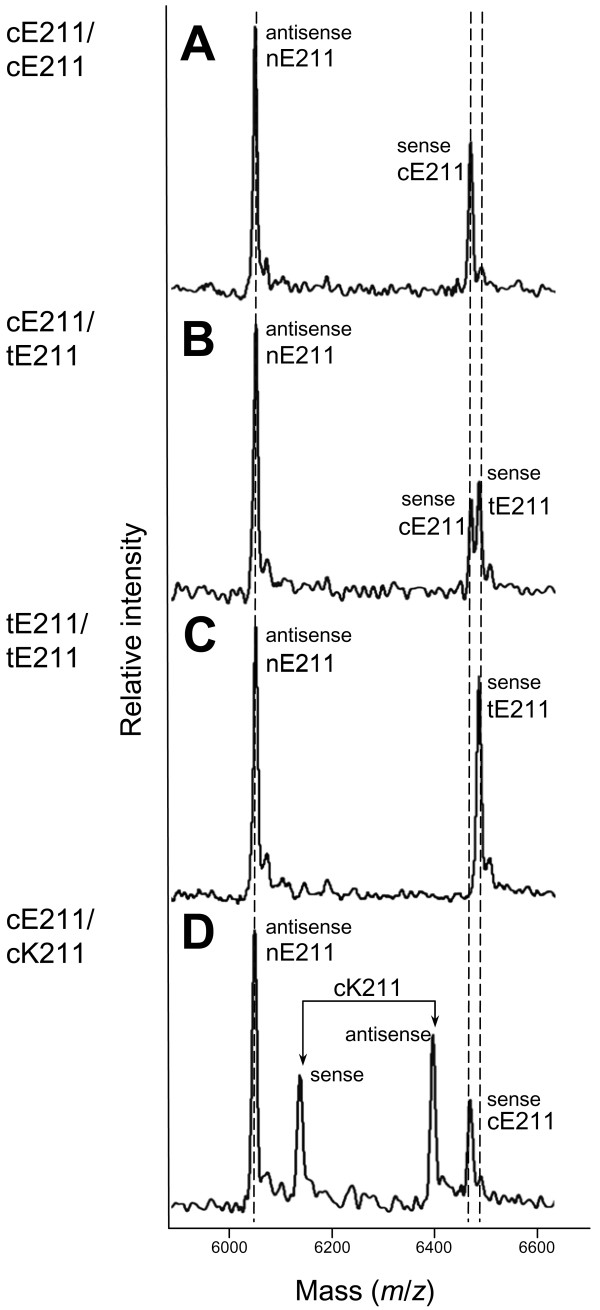
**Mass spectrograms of *Prnp *genotypes for SNPs at codons 210 and 211 in U.S. cattle**. Spectral peaks represent singly-charged ions whose mass-to-charge ratio (*m*/*z*) was compared with calibrants for mass determination. Panels A, B, and C: Representative mass spectrograms showing the three haplotype combinations observed in U.S. cattle in their order of prevalence: cE211/cE211, cE211/tE211, and tE211/tE211. The "antisense nE211" designation refers to a peak generated by either a cE211 or a tE211 allele because the genotype for this analyte is ambiguous in the antisense direction. Although conservative hME assay designs include 40 Dalton spacing between analytes, only 15 Dalton spacing was possible in this assay design. Nevertheless, the typical instrument resolution of approximately 4 Daltons is sufficient to resolve the analytes. Panel D: Mass spectrogram of the diplotype reconstituted from cloned cDNA of the 2006 Alabama BSE case.

Because the Alabama BSE case was reported to be a "deep red, crossbred beef cow...possibly crossed with a Santa Gertrudis or similar breed" [25], additional emphasis was placed on collecting cattle with *Bos indicus *germplasm. The Brahman, Nelore, and Mini-Zebu breeds sampled are expected to contain the most *Bos indicus *germplasm, whereas the Brangus, Beefmaster, Santa Gertrudis, Brahmousin, and Senepol breeds are composite breeds with both *Bos indicus *and *Bos taurus *germplasm. Approximately 48% of the cattle samples from processing plants were from lots where the average *Bos indicus *germplasm composition of animals was estimated by observers to be more than 50%. Of the registered purebred sires and dams tested, about 30% (652) were *Bos indicus *or composites thereof. The K211 allele was not detected in any of the 6062 cattle tested, including those from five commercial beef processing plants in three states (3892 carcasses) and 2170 registered cattle from 42 breeds (Table 2). MALDI-TOF MS diplotypes were unambiguously inferred for all 6062 cattle genomic DNAs and fell into three classes: cE211/cE211, cE211/tE211, and tE211/tE211. A fourth diplotype, cE211/cK211, was observed when recombinant cDNA from the Alabama BSE case was used to reconstitute its diplotype (Figure 2). The upper bounds for prevalence of *Prnp *K211 was estimated to be less than 1 in 2000 cattle based on the 95% quantile of a beta posterior distribution conditional on the observation of 6062 diverse U.S. cattle without any *Prnp *K211 alleles and a conservative uniform prior distribution (Additional file 2). Changing the two non-negative shape parameters, alpha and beta, to that of a more realistic but less conservative probability density function (e.g., alpha = 2 and beta = 5) did not affect the outcome.

**Table 2:** *Prnp *haplotype allele frequencies for codons 210 and 211

		Allele frequencies^a^	
Cattle tested	Animals	cE211	tE211
**Total**	6062	0.984	0.016
**Cattle from commercial beef processing plants**	3892	0.982	0.018
California (n = 1)	392	0.980	0.020
Nebraska (n = 2)	1615	0.999	0.001
Texas, panhandle (n = 1)	588	0.959	0.041
Texas, south (n = 1)	1297	0.971	0.029
**Registered purebred cattle**	2170	0.987	0.013
**From cow-calf herds**	714	0.994	0.006
Angus (7 herds, 4 midwestern states)	330	0.997	0.003
Santa Gertrudis (1 Texas herd)	384	0.991	0.009
**From cattle with diverse pedigrees**	1456	0.984	0.016
Angus	60	1.000	0.000
Ankole-Watusi	30	1.000	0.000
Ayrshire	27	1.000	0.000
Beefmaster	32	1.000	0.000
Belgian Blue	26	1.000	0.000
Blonde D'Aquitaine	24	1.000	0.000
Brahman	59	0.856	0.144
Brahmousin	24	0.979	0.021
Brangus	27	0.933	0.067
Braunvieh	29	1.000	0.000
Brown Swiss	30	1.000	0.000
Charolais	39	1.000	0.000
Chianina^b^	27	1.000	0.000
Corriente	30	1.000	0.000
Gelbvieh	41	1.000	0.000
Guernsey	30	1.000	0.000
Hereford	35	1.000	0.000
Highland	24	1.000	0.000
Holstein	190	1.000	0.000
Jersey	39	1.000	0.000
Limousin	31	1.000	0.000
Maine-Anjou	29	1.000	0.000
Marchigiana	39	1.000	0.000
Mini-Hereford	24	1.000	0.000
Mini-Zebu	27	0.926	0.074
Montbeliard	24	1.000	0.000
Murray Grey	22	1.000	0.000
Nelore	24	0.750	0.250
Piedmontese	22	1.000	0.000
Pinzgauer	24	1.000	0.000
Red Angus	30	1.000	0.000
Red Poll	24	1.000	0.000
Romagnola	26	1.000	0.000
Salers	29	1.000	0.000
Santa Gertrudis	51	0.951	0.049
Senepol	25	0.940	0.060
Shorthorn	29	1.000	0.000
Simmental	41	1.000	0.000
Tarentaise	28	1.000	0.000
Texas Longhorn	28	1.000	0.000
Tuli	33	1.000	0.000
Wagyu	23	1.000	0.000

^a^K211 alleles were not detected in any cattle tested here. Abbreviations for the haplotype sequences of codons 210 and 211: cE211, acC-Gaa; tE211, acT-Gaa.

^b^Mixture of 100% Chianina and Chi-Angus.

### Additional unrecognized *Prnp *sequence variation in U.S. cattle

Because unrecognized DNA sequence variation near *Prnp *codon 211 may interfere with genotype scoring, it was important to sequence the complete *Prnp *coding sequence in diverse sires and dams from purebred collections. Analysis of *Prnp *coding sequences from more than 1400 diverse purebred cattle from 42 breeds identified three previously unreported polymorphisms. All three single nucleotide polymorphisms (SNPs) were in intron 2 at -403, -399 and -276 bp upstream of the *Prnp *start codon, respectively (Figure 1B). A single Guernsey was heterozygous for the SNP at -403, whereas two Brangus and one Brahmousin were heterozygous for the SNP at -399. A number of Beefmaster and Brahman animals contained the minor allele for the SNP at -276. However, neither of these three SNPs nor the 33 other known polymorphisms in this region interfered with scoring E211 or K211 alleles. In addition, 100% of the genotypes from codons 210 and 211 were concordant with those from the MALDI-TOF MS assays (data not shown). The bovine *Prnp *sequence variants, breed-based allele frequencies, animal genotypes, and electropherograms are publicly available [26].

## Discussion

Bovine carriers of the *Prnp *K211 allele may be at risk for developing atypical BSE without exposure to other infectious prion agents (unpublished results, J.A. Richt and S.M. Hall). The present study describes a MALDI-TOF MS assay and synthetic DNA controls that facilitate screening large numbers of cattle for the K211 allele. Our aim was to find the K211 allele – if possible. Consequently, the sampling strategy for commercial and purebred sources was intentionally biased towards *Bos indicus *germplasm because the Alabama BSE case was reported to be of this type. The *Bos indicus *germplasm samples included more than 650 full blood and purebred animals from *Bos indicus *and *Bos indicus *composite breeds. Moreover, a large sampling of Texas beef processing plants increased this *Bos indicus *bias. Another intentional sampling bias was selecting more than 1400 diverse elite registered full blood animals from 42 breeds. The top 17 of these breeds represent greater than 99% of the cattle germplasm used in the U.S., based on the number of their registered progeny [27]. Because the germplasm of elite full blood animals is the foundation of seed stock and commercial cattle in the U.S., it represents a more thorough sampling of U.S. bovine germplasm than a random sample. In spite of the biased sampling, K211 carriers were not detected in any cattle tested. These results indicate, like atypical BSE, the *Prnp *K211 allele is vanishingly rare among U.S. cattle.

The origin of the K211 allele from the Alabama BSE case remains unknown. None of its ancestors were traceable and thus they were not available for testing [21]. The question remains, what was the origin of this *Prnp *K211 allele? Possibilities include: 1) it was a mutation that arose independently in early embryonic development of the 2006 Alabama BSE case, 2) it was a mutation that arose independently in a gamete of one of its parents, 3) it was a mutation that arose recently in a line of cattle whose descendants include the Alabama BSE case, or 4) it was an allele present in a population or breed not tested. Given the apparent scarcity of carriers in the U.S. cattle population, strategies for identifying K211 carriers might include sampling cattle from geographic areas relevant to the Alabama BSE case and the continued sampling of diverse purebred sires and dams from previously untested breeds. Regardless of the origin of the *Prnp *K211 allele from the Alabama BSE case, there is always a remote chance that a new K211 mutation may arise independently in any calf.

In spite of the homology with the pathogenic K200 allele in humans, the functional significance of the K211 allele from the Alabama BSE case remains unknown. A high-throughput DNA test will be essential for identifying carriers needed for future studies designed to test the influence of *Prnp *K211 on development of atypical BSE. A 2006-born heifer calf from the Alabama BSE case is the only known living descendent; however its *Prnp *codon 211 genotype has not yet been reported. Of the 30 or so atypical BSE cases reported around the world, only the Alabama atypical BSE case has been reported to have a K211 allele (unpublished results, J.A. Richt and S.M. Hall). Thus, if additional research shows that the K211 allele causes atypical BSE, it would not be the only cause. A recent report showed that five of six other atypical BSE cases shared a relatively uncommon haplotype that is distinct from those of the Alabama BSE case [17]. Regardless of the type, identifying undesirable bovine *Prnp *alleles provides the opportunity to manage them before they cause disease.

## Conclusion

No groups or breeds of U.S. cattle are presently known to harbor the *Prnp *K211 variant. Because a carrier was not detected, the number of additional atypical BSE cases with K211 will also be vanishingly low.

## Methods

### Cattle samples

Bovine samples from large commercial beef processing plants consisted of approximately 50% from muscle (longissimus dorsi) collected in the winter of 2005–2006, and 50% from whole blood collected in the spring of 2007. The five beef processing plants were located in three states but receive cattle from all over the continental U.S. Registered purebred cattle samples consisted of those from cow-calf herds and diverse sire and dam collections. The Angus cow-calf herd samples were from seven herds and four Midwestern states [28] whereas the Santa Gertrudis cow-calf herd samples were from a single Texas herd.

Samples of male and female registered purebred cattle with diverse pedigrees were taken from semen, blood, or tail hair follicles, depending on gender and availability. Semen from sires used in artificial insemination (AI sires) was obtained from commercial and private suppliers. Where possible, animals within breed were chosen so they did not share parents or grandparents. To estimate the relative diversity among animals in breed groups, the average relationships were computed with CFC version 1.0 (Coancestry, Inbreeding (F), Contribution) [29]. For breed groups where pedigrees were readily available, 24 animals with four-generation pedigrees were evaluated. The average numerator relationships (i.e., average coancestries) among groups of 24 purebred or full blood animals were: Angus, 0.057; Beefmaster, 0.122; Brahman, 0.047; Brangus, 0.056; Charolais, 0.054; Corriente, 0.062; Gelbvieh, 0.058; Hereford, 0.045; Murray Grey, 0.047; Nelore, 0.079; Red Angus, 0.047; Senepol, 0.060; Simmental, 0.055; Tarentaise, 0.073; and Wagyu, 0.114.

Beef breed collections that were composed entirely of AI sires included: Angus, Beefmaster, Blonde D'Aquitaine, Brahman, Braunvieh, Charolais, Gelbvieh, Hereford, Limousin, Maine-Anjou, Nelore, Red Angus, Salers, Santa Gertrudis, Shorthorn, Simmental, and Texas Longhorn. Beef breeds where tissues from some females were collected included: Ankole-Watusi, Belgian Blue, Brahmousin, Brangus, Chianina, Corriente, Highland, Marchigiana, Mini-Hereford, Mini-Zebu, Murray Grey, Piedmontese, Pinzgauer, Red Poll, Romagnola, Senepol, Tarentaise, Tuli, and Wagyu.

Dairy breed collections that were composed entirely of AI sires included: 85 Holsteins, 7 Jerseys, 2 Brown Swiss, and 2 Guernsey (Beltsville Agricultural Research Center [BARC] Dairy Cattle Panel version 1.0). Selection criteria for this group included sire prominence (i.e., high usage in the dairy industry) and diversity. This set was complemented with liver samples from market dairy cattle [30] and various semen or hair samples available from willing private owners of registered purebred cattle including: Ayrshire, Brown Swiss, Guernsey, Holstein, Jersey, and Montbeliard. No experiments were performed on any of these animals for this research. All cattle samples were collected during the normal processes of working, showing, or federally inspected processing of animals.

### DNA extraction and sequencing

DNA from muscle and whole blood samples was extracted by use of a solid-phase system incorporating either spin-columns or 96-well microtitration plates according to the manufacturer's instructions (Gentra Systems, Inc., Minneapolis, MN, USA). DNA from liver, muscle skin, or hair samples was extracted by standard procedures. Briefly, minced tissue (35 mg) was suspended in a lysis solution of 2.5 ml 10 mM TrisCl, 400 mM NaCl, 2 mM EDTA, 1% wt/vol sodium dodecyl sulfate, RNase A (250 ug/ml; Sigma Chemical Co., St. Louis, MO, USA), pH 8.0. The solution was incubated at 37°C with gentle agitation. After 1 hour, 1 mg proteinase K was added (Sigma Chemical Co.) and the solution was incubated overnight at 37°C with continued agitation. The sample was extracted twice with 1 vol of phenol:chloroform:isoamyl alcohol (25:24:1), and once with 1 vol of chloroform before precipitation with 2 vol of 100% ethanol. The precipitated DNA was washed once in 70% ethanol, briefly air dried, and dissolved in a solution of 10 mM TrisCl, 1 mM EDTA (pH 8.0). DNA from bull semen was extracted similarly with slight modification, including the presence of 40 mM dithiothreitol [31].

Polymerase chain reaction (PCR) cocktails and DNA sequencing reactions were carried out as previously described [22,23]. Following exonuclease I digestion, the amplicons were sequenced with BigDye terminator chemistry on an ABI 3730 capillary sequencer (PE Applied Biosystems, Foster City, CA, USA). Oligonucleotide primers were designed to amplify 1498 bp and 935 bp amplicons in separate reactions (Figure 1C). The 935 bp amplicon was contained within the 1498 bp amplicon and each contained the entire 795 bp *Prnp *coding sequence (with six octapeptide repeats). Oligonucleotide primers for amplification were chosen so that they were not overlaying known SNPs or bovine repetitive elements. Both strands of each amplicon were sequenced for each animal to increase the quality of their consensus sequence and assist in recognizing "allelic drop out" due to misamplification. This phenomenon commonly occurs when an individual's genome contains a previously unrecognized polymorphism in the binding site of the amplification primer. The sequence mismatch between the amplification primer and the genomic DNA reduces the stability of heteroduplex formation often results in allele misamplification (sometimes referred to as null alleles). The DNA sequences, allele frequencies, SNP genotypes of animals, and their tracefiles are publicly available [26].

### MALDI-TOF MS genotyping of adjacent SNPs

The hME chemistry (Sequenom, Inc., San Diego, CA, USA) was used to genotype the adjacent SNPs in *Prnp *codons 210 and 211 from both sense and antisense DNA strands (Table 1). Unlike single-base extension chemistries that use all four dideoxynucleotides together, hME chemistry uses selected combinations of dideoxynucleotides and deoxynucleotides and allows for multiple base extension across adjacent SNPs. The result may be used to unambiguously infer haplotypes for multiple, sequential SNPs. The assay was designed to score both DNA strands from the same PCR reaction for each animal. Because each genotype result must agree, this strategy provides an internal control for error checking and increases the confidence of detecting a rare allele. A 203 bp region, approximately centered about codon 211, was chosen for PCR amplification (Figure 1C). The amplification primer binding sites for this amplicon are not known to be polymorphic in any cattle population. Additional non-specific sequences (i.e., mass tags) were added to the hME amplification primers to increase their mass and thereby shift them out of the useful region of the MALDI-TOF mass spectrum. The amplification primers with these mass tags produced a 226 bp amplicon for genetic analysis (Table 1). For some DNA samples, particularly those from hair follicles, success in amplification was influenced by the type of Taq polymerase used. Results presented here were produced with Thermo-Start^® ^PCR Master Mix (ABgene USA, Rochester, NY, USA). After PCR, a few microliters of each reaction were analyzed by agarose gel electrophoresis to monitor the amplification results. Shrimp alkaline phosphatase enzyme was subsequently added according to manufacturer's instruction to convert unincorporated dNTPs to dNDPs so they do not interfere with subsequent reactions. Each sample was split into two hME reactions containing different termination mixtures (Table 1, ddA or ddC/ddG) for the respective sense and antisense reactions. After thermocyling, the hME reactions of the paired samples were either reconstituted to conserve reagents and increase MALDI-TOF MS throughput or processed individually. A cation-exchange resin was added to remove salts that may interfere with analysis by mass spectroscopy. Samples were spotted in nanoliter amounts onto a matrix-arrayed silicon chip with 384 elements and analyzed with the manufacturer's MassARRAY compact system and software (Sequenom, Inc.). Synthetic DNA controls (42 bp) were designed to be nested within the 226 bp hME PCR product to minimize the potential amplification of any cross-contamination between synthetic K211 alleles and animal samples.

### Diplotype reconstitution of the Alabama BSE case with recombinant cDNA

Because genomic DNA from the 2006 Alabama BSE case was not available, its diplotype was reconstituted from recombinant cDNA clones. *Prnp *cDNA from each allele was cloned into a plasmid vector as previously described (unpublished results, J.A. Richt and S.M. Hall). To minimize the chance of contaminating cattle DNAs derived from field samples with that derived from the Alabama BSE case, plasmid cDNA from the Alabama BSE case was not directly used in MALDI-TOF MS genotyping. Instead, the purified plasmid DNAs from the respective recombinant cDNA clones were used in nanogram amounts as templates in separate reactions to synthesize a 141 bp PCR fragment with amplification primers: 5'-tgtgcatgactgtgtcaa-3' (59311, sense) and 5'-gattctctctggtactggg-3' (59311, antisense). Like the 42 bp synthetic DNA controls, this 141 bp fragment was also designed to be nested within the 203 bp region routinely amplified for MALDI-TOF MS genotyping to minimize the possibility of false positives due to PCR amplification of small amounts of any contaminating K211 cDNA. The respective 141-bp PCR products derived from the Alabama BSE case were pooled, precipitated in 2 vol of 100% ethanol, washed in 70% ethanol, briefly air dried, and dissolved in a solution of 10 mM TrisCl, 1 mM EDTA (pH 8.0). Equal amounts of the two preparations were mixed and used in hME reactions as described in Methods section "MALDI-TOF MS genotyping of adjacent SNPs."

### Estimating the prevalence of *Prnp *K211 carriers

Prevalence may be defined as the proportion of *Prnp *K211 carrier animals in the U.S. cattle population. Although this could be estimated directly by calculating a simple proportion (e.g., one K211 carrier per 6062 cattle tested), the animals from beef processing facilities were not randomly sampled and the diverse registered AI sires contribute a disproportionate number of alleles to U.S. cattle populations. Thus, prevalence was estimated with a posterior distribution given the number of observed carriers, the number of animals sampled, and a prior distribution that reflected prior knowledge and uncertainty. The beta distribution was used as a prior distribution for binomial proportions in Bayesian analysis [32]. The beta family of distributions was used for the posterior and prior distributions because they are conjugate (i.e., they yield the same functional forms for both prior and posterior distributions). The uniform distribution, a member of the beta family, was used as the prior distribution because it is conservative and does not underestimate the frequency of *Prnp *K211 carriers. The prior knowledge was that both the prevalence of atypical BSE and *Prnp *K211 carriers are low based on previous BSE surveillance and prion gene DNA sequencing, respectively [22-24,33]. All values of prevalence are equally likely under the prior uniform distribution; hence, posterior distribution with a uniform prior is biased to the high side yielding an inflated (i.e., conservative) 95% quantile. The posterior distribution for the prevalence of K211 carriers with a uniform prior was modeled by the beta distribution as follows:

$$f(x;\alpha ,\beta )=\frac{\Gamma (\alpha +\beta )}{\Gamma (\alpha )\Gamma (\beta )}x^{\alpha -1}\cdot {\left(1-x\right)}^{\beta -1}$$

where *x *is the prevalence of carriers (heterozygotes), α is the number of carriers observed plus 1, and β is the total number of animals sampled plus 1. The 95% quantile of *f *was computed using the BETAINV function of Microsoft Office Excel which returns the inverse of the cumulative beta probability density function (BETADIST).

## Competing interests

All authors, except TSK, declare that they have no competing interests. TSK is the President and principal owner of Intrepid Bioinformatics Solutions, Inc., a company that develops data brokerage systems in support of life sciences research.

## Authors' contributions

MPH conceived the experiment, designed sampling and collection of tissues from registered purebred beef cattle, designed genotyping assays and DNA sequencing, analyzed results, and prepared the manuscript. JWK and GPH participated in computational analysis of genotypes and DNA sequences. JAR provided unpublished sequence, results, and the cloned bovine E211K cDNA controls. MK, TLW, and SDS carried out the organization and collection of commercial cattle samples from beef processing facilities. EC and DAK carried out the collection and extraction of samples from beef processing facilities. TSS, CPVT, HLN, and CCC carried out design, collection, and extraction of purebred dairy, Wagyu, and Senepol cattle samples, respectively. TSK carried out the design, development, and transfer of the *Prnp *trace data and diplotypes to the public website. TPLS supervised the DNA sequencing and participated in manuscript editing. MLC and WWL participated in experimental design, results analysis, and manuscript editing. All authors have read and approved the final manuscript.

## Supplementary Material

Additional file 1 **Depictions of mass spectrograms for all ten possible paired haplotype combinations of synthetic DNA control templates for SNPs at codons 210 and 211**. Depictions of spectral peaks represent singly-charged ions whose mass-to-charge ratio (*m/z*) was compared with calibrants for mass determination. The "antisense nE211" designation refers to a peak generated by either a cE211 or a tE211 allele because the genotype for this analyte is ambiguous in the antisense direction. Click here for file

Additional file 2 Estimating the 95% quantile for prevalence of *Prnp *K211 carriers. Click here for file

## References

[B1] Anderson RM, Donnelly CA, Ferguson NM, Woolhouse MEJ, Watt CJ, Udy HJ, MaWhinney S, Dunstan SP, Southwood TRE, Wilesmith JW, Ryan JB, Hoinville LJ, Hillerton JE, Austin AR, Wells GA (1996). Transmission dynamics and epidemiology of BSE in British cattle. Nature.

[B2] Wells GA, Scott AC, Johnson CT, Gunning RF, Hancock RD, Jeffrey M, Dawson M, Bradley R (1987). A novel progressive spongiform encephalopathy in cattle. Vet Rec.

[B3] Prusiner SB (1997). Prion diseases and the BSE crisis. Science.

[B4] Wilesmith JW, Ryan JB, Atkinson MJ (1991). Bovine spongiform encephalopathy: epidemiological studies on the origin. Vet Rec.

[B5] The World Organisation for Animal Health, O I E:  Number of reported cases of bovine spongiform encephalopathy (BSE) in farmed cattle worldwide. http://www.oie.int/eng/info/en_esbmonde.htm.

[B6] Bruce ME, Will RG, Ironside JW, McConnell I, Drummond D, Suttie A, McCardle L, Chree A, Hope J, Birkett C, Cousens S, Fraser H, Bostock CJ (1997). Transmissions to mice indicate that 'new variant' CJD is caused by the BSE agent. Nature.

[B7] Collinge J, Rossor M (1996). A new variant of prion disease. Lancet.

[B8] Hill AF, Desbruslais M, Joiner S, Sidle KCL, Gowland I, Collinge J, Doey LJ, Lantos P (1997). The same prion strain causes vCJD and BSE. Nature.

[B9] Will RG, Ironside JW, Zeidler M, Cousens SN, Estibeiro K, Alperovitch A, Poser S, Pocchiari M, Hofman A, Smith PG (1996). A new variant of Creutzfeldt-Jakob disease in the UK. Lancet.

[B10] The National Creutzfeldt-Jakob Disease Surveillance Unit: CJD Statistics. http://www.cjd.ed.ac.uk/figures.htm.

[B11] Biacabe AG, Laplanche JL, Ryder S, Baron T (2004). Distinct molecular phenotypes in bovine prion diseases. EMBO Rep.

[B12] Biacabe AG, Morignat E, Vulin J, Calavas D, Baron T (2008). Atypical bovine spongiform encephalopathies, France, 2001–2007. Emerg Infect Dis.

[B13] Capobianco R, Casalone C, Suardi S, Mangieri M, Miccolo C, Limido L, Catania M, Rossi G, Di Fede G, Giaccone G, Bruzzone MG, Minati L, Corona C, Acutis P, Gelmetti D, Lombardi G, Groschup MH, Buschmann A, Zanusso G, Monaco S, Caramelli M, Tagliavini F (2007). Conversion of the BASE prion strain into the BSE strain: the origin of BSE?. PLoS Pathog.

[B14] Casalone C, Zanusso G, Acutis P, Ferrari S, Capucci L, Tagliavini F, Monaco S, Caramelli M (2004). Identification of a second bovine amyloidotic spongiform encephalopathy: molecular similarities with sporadic Creutzfeldt-Jakob disease. Proc Natl Acad Sci U S A.

[B15] Brown P, McShane LM, Zanusso G, Detwile L (2006). On the question of sporadic or atypical bovine spongiform encephalopathy and Creutzfeldt-Jakob disease. Emerg Infect Dis.

[B16] Polak MP, Zmudzinski JF, Jacobs JG, Langeveld JP (2007). Atypical status of bovine spongiform encephalopathy in Poland: a molecular typing study. Arch Virol.

[B17] Clawson ML, Richt JA, Baron T, Biacabe AG, Czub S, Heaton MP, Smith TP, Laegreid WW (2008). Association of a bovine prion gene haplotype with atypical BSE. PLoS ONE.

[B18] Kovacs GG, Puopolo M, Ladogana A, Pocchiari M, Budka H, van Duijn C, Collins SJ, Boyd A, Giulivi A, Coulthart M, Delasnerie-Laupretre N, Brandel JP, Zerr I, Kretzschmar HA, de Pedro-Cuesta J, Calero-Lara M, Glatzel M, Aguzzi A, Bishop M, Knight R, Belay G, Will R, Mitrova E (2005). Genetic prion disease: the EUROCJD experience. Hum Genet.

[B19] Goldgaber D, Goldfarb LG, Brown P, Asher DM, Brown WT, Lin S, Teener JW, Feinstone SM, Rubenstein R, Kascsak RJ, Boellaard JW, Gajdusek DC (1989). Mutations in familial Creutzfeldt-Jakob disease and Gerstmann-Straussler-Scheinker's syndrome. Exp Neurol.

[B20] Lee HS, Sambuughin N, Cervenakova L, Chapman J, Pocchiari M, Litvak S, Qi HY, Budka H, del Ser T, Furukawa H, Brown P, Gajdusek DC, Long JC, Korczyn AD, Goldfarb LG (1999). Ancestral origins and worldwide distribution of the PRNP 200K mutation causing familial Creutzfeldt-Jakob disease. Am J Hum Genet.

[B21] United States Department of Agriculture, Animal Plant Health Inspection Service: Alabama BSE investigation final epidemiology report May 2, 2006. http://www.aphis.usda.gov/newsroom/hot_issues/bse/downloads/EPI_Final5-2-06.pdf.

[B22] Clawson ML, Heaton MP, Keele JW, Smith TPL, Harhay GP, Laegreid WW (2006). Prion gene haplotypes of U.S. cattle. BMC Genet.

[B23] Heaton MP, Leymaster KA, Freking BA, Hawk DA, Smith TPL, Keele JW, Snelling WM, Fox JM, Chitko-McKown CG, Laegreid WW (2003). Prion gene sequence variation within diverse groups of U.S. sheep, beef cattle, and deer. Mamm Genome.

[B24] Seabury CM, Honeycutt RL, Rooney AP, Halbert ND, Derr JN (2004). Prion protein gene (PRNP) variants and evidence for strong purifying selection in functionally important regions of bovine exon 3. Proc Natl Acad Sci U S A.

[B25] United States Department of Agriculture, Animal Plant Health Inspection Service:  Second USDA confirmatory test results positive for BSE March 15, 2006. http://www.usda.gov/wps/portal/usdahome?contentidonly=true&contentid=2006/03/0090.xml.

[B26] A SNP Marker Set for DNA-based Traceback in North American Beef and Dairy Cattle. http://cgemm.louisville.edu/usmarc/MARC_web_page/traceback.html.

[B27] Heaton MP, Chitko-McKown CG, Grosse WM, Keele JW, Keen JE, Laegreid WW (2001). Interleukin-8 haplotype structure from nucleotide sequence variation in commercial populations of U.S. beef cattle. Mamm Genome.

[B28] Laegreid WW, Heaton MP, Keen JE, Grosse WM, Chitko-McKown CG, Smith TP, Keele JW, Bennett GL, Besser TE (2002). Association of bovine neonatal Fc receptor alpha-chain gene (FCGRT) haplotypes with serum IgG concentration in newborn calves. Mamm Genome.

[B29] Sargolzaei M, Iwaisaki H, Colleau JJ (2006). CFC:  A tool for monitoring genetic diversity: August 13-18; Belo Horizonte, MG, Brazil..

[B30] Heaton MP, Keen JE, Clawson ML, Harhay GP, Bauer N, Shultz C, Green BT, Durso L, Chitko-McKown CG, Laegreid WW (2005). Use of bovine single nucleotide polymorphism markers to verify sample tracking in beef processing. J Am Vet Med Assoc.

[B31] Heaton MP, Grosse WM, Kappes SM, Keele JW, Chitko-McKown CG, Cundiff LV, Braun A, Little DP, Laegreid WW (2001). Estimation of DNA sequence diversity in bovine cytokine genes. Mamm Genome.

[B32] Evans M, Hastings N, Peacock B (2000). Beta Distribution. Statistical Distributions.

[B33] United States Department of Agriculture, Animal Plant Health Inspection Service: An Estimate of the prevalence of BSE in the United States. http://www.aphis.usda.gov/newsroom/hot_issues/bse/downloads/BSEprev-estFINAL_7-20-06.pdf.

