# Supplementary figures and images for: Prevalence of the prion protein gene E211K variant in U.S. cattle
Source: BMC Vet Res. 2008 Jul 14;4:25. doi: 10.1186/1746-6148-4-25 (PMC2478677; doi:10.1186/1746-6148-4-25)

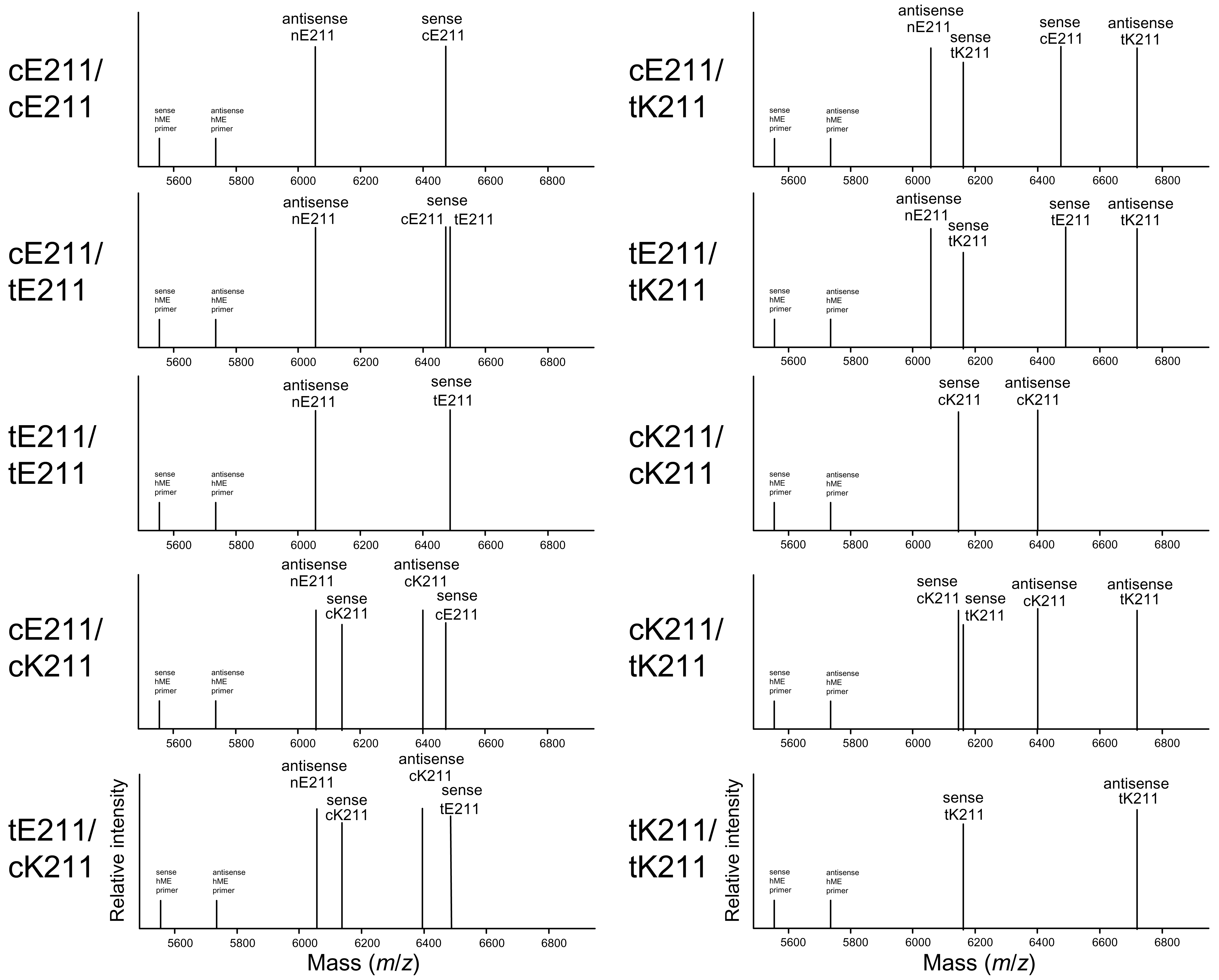

Supplement: Additional file 1 — Depictions of mass spectrograms for all ten possible paired haplotype combinations of synthetic DNA control templates for SNPs at codons 210 and 211. Depictions of spectral peaks represent singly-charged ions whose mass-to-charge ratio (m/z) was compared with calibrants for mass determination. The "antisense nE211" designation refers to a peak generated by either a cE211 or a tE211 allele because the genotype for this analyte is ambiguous in the antisense direction. [file 1746-6148-4-25-S1.jpeg]
